# Reduced microbiome alpha diversity in young patients with ADHD

**DOI:** 10.1371/journal.pone.0200728

**Published:** 2018-07-12

**Authors:** Alexander Prehn-Kristensen, Alexandra Zimmermann, Lukas Tittmann, Wolfgang Lieb, Stefan Schreiber, Lioba Baving, Annegret Fischer

**Affiliations:** 1 Department of Child and Adolescent Psychiatry and Psychotherapy, Centre for Integrative Psychiatry, University Hospital Schleswig-Holstein, Kiel, Germany; 2 Institute of Clinical Molecular Biology, University Hospital Schleswig-Holstein, Kiel, Germany; 3 Institute for Epidemiology, University Hospital Schleswig-Holstein, Kiel, Germany; 4 Clinic of Internal Medicine I, University Hospital Schleswig-Holstein, Kiel, Germany; Kunming Institute of Zoology, Chinese Academy of Sciences, CHINA

## Abstract

ADHD is a psychiatric disorder which is characterized by hyperactivity, impulsivity and attention problems. Due to recent findings of microbial involvement in other psychiatric disorders like autism and depression, a role of the gut microbiota in ADHD pathogenesis is assumed but has not yet been investigated. In this study, the gut microbiota of 14 male ADHD patients (mean age: 11.9 yrs.) and 17 male controls (mean age: 13.1 yrs.) was examined via next generation sequencing of 16S rDNA and analyzed for diversity and biomarkers. We found that the microbial diversity (alpha diversity) was significantly decreased in ADHD patients compared to controls (p_Shannon_ = 0.036) and that the composition (beta diversity) differed significantly between patients and controls (p_ANOSIM_ = 0.033, p_ADONIS_ = 0.006, p_betadisper_ = 0.002). In detail, the bacterial family *Prevotellacae* was associated with controls, while patients with ADHD showed elevated levels of *Bacteroidaceae*, and both *Neisseriaceae* and *Neisseria spec*. were found as possible biomarkers for juvenile ADHD. Our results point to a possible link of certain microbiota with ADHD, with *Neisseria spec*. being a very promising ADHD-associated candidate. This finding provides the basis for a systematic, longitudinal assessment of the role of the gut microbiome in ADHD, yielding promising potential for both prevention and therapeutic intervention.

## Introduction

With a worldwide prevalence of 3–5% [1, 2], ADHD is one of the most commonly diagnosed psychiatric diseases in childhood and adolescence. ADHD is characterized by symptoms of inattention, hyperactivity, and/or impulsivity [3]. The symptoms are caused by dysfunctions in the dopaminergic neurotransmitter system [4–6] and fronto-striatal brain functions [7–11]; however, the definitive pathogenesis of ADHD remains elusive due to its complex, multifactorial nature. Besides a significant genetic vulnerability [12, 13], external factors, such as perinatal conditions (e.g. low birth weight, prematurity, prenatal exposure to alcohol and/or toxins originating from smoking cigarettes) [14, 15] or socioemotional environment during postnatal development [16, 17], and also food constituents and micronutrients have an impact on ADHD symptom severity [18–22]. Food intolerances are often related to gastrointestinal immune dysregulation which can result in chronic inflammations. In turn, food intolerances and chronic inflammations are increasingly suspected in ADHD [23, 24], leading to the assumption that gastrointestinal dysregulation may be involved in ADHD [25].

It is well known that gut microbiota and the central nervous system are interconnected in a bidirectional fashion, termed the *gut-brain axis*. Recent studies underline the importance of the human gut microbiome in human health. Not only are gut microbes involved in digestion, metabolism and weight control [26–28], they are also potent stimulants for the human immune system [29]. There is growing appreciation for the fact that the gut microbiome might also be involved in human psychopathology [30–34]. As an interface with the environment, the gut microbiota is prone to environmental influences [35]. Disturbances in early microbiome development have a severe impact on the development of a healthy immune system, elevating, for example, the risk of atopic diseases [36]. Many of the risk factors associated with ADHD, such as delivery method, gestational age, type of feeding, maternal health, and early life stressors, have an effect on the microbiota [37, 38]. Regarding the gut-brain axis and the influence of the microbiota on the CNS, it is conceivable that a disturbance in a child´s early microbiota may change the gastrointestinal environment, making the organism prone to psychiatric disorders.

Several psychiatric disorders like stress responsivity, anxiety-like behaviors, sociability, and cognition [39–41], as well as anxiety, depression, and autism [42, 43] have already been linked to changes in microbial communities. In addition, experiments with germ-free mice have yielded promising results, suggesting an influence of the microbiota on the activity level and pointing to a possible link of the microbiome to hyperactivity disorders like ADHD [44]. One study suggested that the composition of some gut microbiota at some points in time was reduced in toddlers who later exhibited neurodevelopmental disorders (also including ADHD) [45]. However, it is still unclear whether juvenile ADHD is accompanied by alterations in the human microbiome, e.g. in the microbial diversity. The diversity of microbes within a given body habitat can be described by the richness and evenness, i.e.by the number of species in relation to the species’ abundance within a sample (alpha-diversity), with a high diversity being linked to a healthy state [46, 47]. Therefore, we assume that young patients with ADHD display reduced diversity and differ in microbial composition when compared to healthy controls.

## Material and methods

### Study participants

Fourteen male children and adolescents with ADHD (M = 11.9 yrs., SD = 2.5) and 17 controls (M = 13.1 yrs., SD = 1.7) participated in this study. Patients and controls did not differ in age (p = 0.138), BMI (p = 0.728), or IQ (p = 0.149; see also Table 1). All participants and their parents were Caucasians. All family members were born and raised in Germany (exeept for one father who was of Polish origin) and also currently live in North Germany. In the ADHD families both biological parents of nine patients were present; in one family, only the biological mother together with a stepfather (he was excluded from further analyses), in three families only the biological mother, and in one family only the biological father were present. In control families biological parents of 12 controls were present; in one family the biological mother together with a stepfather (he was excluded from further analyses) and in four families only the biological mothers were present. Chi-square test revealed no group differences regarding the distribution of family structure: χ = 1.29, p = 0.731. The net income per month in ADHD families was less than 2000€ in 3 families and more than 2000€ in 10 families. In controls it was less than 2000€ in 2 families and more than 2000€ in 13 families (three families did not report their income). Fisher´s test showed that the net income reports did not differ between groups: p = 0.639. All participants were asked to indicate on a 4-point scale (1 = never, 2 = once a week, 3 = several times a week, 4 = daily) how often they consumed fast-food, meat/sausages/cold cuts, fruits/vegetables, or yoghurt and other milk products. The Mann-Whitney-U test revealed that patients and controls did not differ with regards to their food habits (p > 0.365, see Table 1).

**Table 1:** Characteristics of participants.

		ADHD		Controls		ADHD vs. Controls	
		Mean	SD	Mean	*SD*	*U*	*p*
Age		11.9	2.5	13.1	1.7	81.5	0.138
IQ		103.8	13.9	110.4	10.9	82.0	0.149
BMI		19.0	3.9	18.0	2.5	103.5	0.728
Eating behavior	Meat/sausages	2.9	0.73	3.2	0.7	95.5	0.356
	Fruits/vegetables	3.5	0.65	3.3	0.6	95.5	0.356
	Yoghurt	3.1	0.83	2.8	0.8	96.5	0.377
	Other milk products	3.5	0.65	3.6	0.5	99.0	0.813
	Fast-food	2.2	0.38	2.2	0.4	101.5	0.711
CBCL	Anxious/depressed	59.1	8.6	52.9	5.2	53.5	**0.008**
	Withdrawn/depressed	58.1	7.6	53.8	6.7	80.0	0.128
	Somatic complaints	58.4	7.5	52.3	4.0	59.0	**0.017**
	Social problems	64.1	11.5	50.7	2.1	31.0	**< 0.001**
	Thought problems	55.2	8.4	51.1	3.0	88.5	0.230
	Attention problems	65.8	8.2	52.0	4.4	10.0	**< 0.001**
	Rule-breaking behavior	60.8	11.1	52.1	4.4	61.5	**0.021**
	Aggressive behavior	62.7	10.0	51.2	3.0	36.0	**0.001**
	Internalizing	59.0	8.1	47.9	9.2	45.0	**0.003**
	Externalizing	60.9	12.4	44.8	7.3	34.5	**< 0.001**
	Total problems	63.9	10.3	44.6	8.5	16.0	**< 0.001**
FBB-HKS	Attention problems	7.3	1.1	3.8	1.9	15.0	**< 0.001**
	Hyperactivity	7.2	1.4	4.5	0.9	12.0	**< 0.001**
	Impulsivity	7.4	1.2	5.1	1.2	25.5	**< 0.001**
	Total	7.4	1.0	3.7	1.6	7.0	**< 0.001**
WURS-K	Fathers	21.2	4.3	16.5	2.2	49.5	0.488
	Mothers	17.5	2.4	8.7	1.1	40.0	**0.003**
ADHD-SB	Fathers	10.2	2.7	8.2	1.7	54.5	0.716
	Mothers	10.8	2.2	4.9	0.8	53	**0.016**

Note: Bold values indicate a significant comparison; U, U-value according to Mann-Whitney-U- test; ADHD, attention-deficit hyperactivity disorder; CBCL, Child Behavior Checklist; FBB-HKS, German ADHD rating scale for children (Fremdbeurteilungsbogen für hyperkinetische Störungen); WURSK, Wender-Utah-Rating Scale; ADHS-SB, German ADHD rating scale for adults (ADHS-Selbstbeurteilung); eating behavior ranged from 1 (never) to 4 (daily).

All children and their parents were interviewed using a German translation of the Revised Schedule for Affective Disorders and Schizophrenia for School-Age Children: Present and Lifetime Version (K-SADS-PL) [48, 49]. Interviews were performed by experienced child and adolescent psychiatrists and psychologists. Standardized questionnaires, the Child Behavior Checklist (CBCL) [50] and the German ADHD rating scale (Fremdbeurteilungsbogen für hyperkinetische Störungen, FBB-HKS) [51], were completed by parents to assess any psychiatric symptoms in their children. According to the DSM-IV-TR, all patients met the criteria for ADHD (12 patients with combined type, two patients with inattentive type; note that even after an exclusion of both patients with the inattentive type, the results of the microbiome analyses reported below remained significant). Six patients additionally fulfilled the criteria for comorbid oppositional defiant disorder (ODD). Ten patients had been taking medicine for more than one year to treat ADHD symptoms (9x Medikinet®, 1x Equasym®). Nine of them followed the instruction to discontinue taking the medicine for at least 48h prior to sample collection.

According to parental ratings, patients displayed more attention problems, hyperactivity, and impulsivity than did controls (all p < 0.001, see Table 1). Controls did not suffer from any psychiatric abnormalities. Parental reports revealed that all participants were free of any neurological, immunological, or endocrinological diseases.

Since ADHD shows high heritability [12, 13], we explored the microbial composition in participants´ parents as well. For this purpose, we screened for possible ADHD symptoms in participants´ parents using self-rating questionnaires: Parents worked on the Wender-Utah-Rating Scale—German short version (WURS-k) [52–54], a self-rating instrument focusing on childhood ADHD psychopathology retrospectively. One mother and three fathers received a sum score of over 30 indicating that childhood ADHD symptoms could have been present in these parents [52, 55]. In addition, parents filled out a short self-rating behavioral questionnaire, the ADHS-SB [56], based on DSM-IV criteria for the assessment of ADHD symptoms. Here, two mothers and three fathers of patients received a sum score above the conservative cut-off of 19 [57].

Patient families were recruited via our out-patient department; families of healthy children were recruited by newspaper announcement. All participating children and their parents gave written, informed consent after the procedures had been fully explained. Families were reimbursed with a voucher for their participation. The study was approved by the ethics committee of the medical faculty of the University of Kiel (Ref.-No. A125/14) and carried out in accordance with the latest version of the Declaration of Helsinki.

### DNA and RNA extraction, sequencing, and processing

Fecal DNA was collected in Sarstedt fecal collection tubes (Nümbrecht, Germany) and stored at 4°C until preparation. Total DNA from fecal samples was extracted using FastDNA^TM^ SPIN KIT FOR SOIL (Qbiogene, Carlsbad, CA, USA) as per the manufacturer protocol after incubation in 200 ml Tris Lysisbuffer and 25ml proteinase K for 2 hours at 56°C. Extracted DNA was stored at -80°C. For sequencing, DNA was amplified using the primer pair 27F-338R for the variable regions V1 and V2. Normalization of PCR products was done with the SequalPrep Normalization Plate Kit (Thermo Fischer Scientific, Waltham, MA, USA), and products were pooled equimolarly for sequencing on the Illumina MiSeq (Illumina Inc., San Diego, CA, USA).

Sequences with a read length less than 200bp and a quality score lower than 25 were rejected. Noise reduction was carried out using Mothur [58, 59]. Ambiguous sequences, sequences with more than eight homopolymers, chimerical sequences, and sequences which differed in the primer or barcode sequence were removed. The output was normalized to 7000 sequences per sample. The sequences were binned into operational taxonomic units (OTUs) with 97% similarity. OTUs are groups of sequences which are clustered based on similarity, allowing taxonomical assignment.

### Alpha/Beta diversity and taxonomic plots

Alpha diversity of the samples was measured by observed species, the Shannon diversity, and the Chao1 index. The observed species index measures the number of different species per sample which is defined as “richness”. The Chao1 index is also a qualitatively measure of alpha diversity which, beside species richness, takes into account the ratio of singletons (n = 1) to doubletons (n = 2) giving more weight to rare species. However, regarding diversity, not only the qualitative amount of species, but also the abundance of the species must be taken into account. The relative abundances of the different species making up the samples’ richness are defined as “evenness”. The Shannon-diversity index relates both, OTU richness and evenness. The association between microbial diversity and ADHD subtypes was tested via multiple linear regression, with microbial alpha diversity as the dependent variable and attention deficits, hyperactivity, and impulsivity as explanatory variables. For this purpose, we used the parental ratings as assessed by the FBB-HKS, since this questionnaire is designed to determine the severity of these three cardinal symptoms according to the DSM-VI [51]. Pairwise comparisons were done using the Wilcoxon rank-sum test for nonparametric data. T-tests were performed after visual data inspection by histograms and when normal distribution of data was given as tested by the Shapiro-Wilk test for normality.

Multivariate statistics were conducted via ANOSIM, ADONIS, and the function “betadisper” from the R package vegan v2.4–1 to analyze microbial beta-diversity which describes the diversity in a microbial community between different samples. ANOSIM is rank-based and tests for similarities, whereas ADONIS tests the homogeneity of dispersion; betadisper tests the similarity of composition among groups. Non-metric multidimensional scaling (NMDS), the most robust, unconstrained and distance-based ordination method, was performed with Bray-Curtis dissimilarity as implemented in the R package vegan v2.4-1. Redundancy analysis is a constrained method based on multiple linear regressions to extract and summarize the variation in a set of response variables which can be explained by a set of explanatory variables. OTU count data were Hellinger-transformed as implemented in the R package vegan v2.4–1. The community composition data matrix that results from deep-sequencing diversity counting is usually characterized by a multitude of zero and single counts of OTUs. To generate data containing many zeros suitable for analysis by linear methods, such as redundancy analysis (RDA), transformation of data like the Hellinger transformation is recommended [60, 61]. Hellinger transformation gives low weights to variables with low counts and many zeros. Contribution of highly correlating OTUs (P_Ord_ < 0.01) with redundancy axes was identified using the envfit functions from the R package vegan [62].

To determine potential biomarker OTUs, which differ in abundance and occurrence between sample groups, full linear discriminant analysis (LDA) effect size (LEfSe) [63] analysis was performed via the Galaxy web application with the Huttenhower lab’s tool. LEfSe analysis finds OTUs or other features which are most likely to explain differences between sample groups. As threshold, a p-value of 0.05 was established [63]. The Kruskal-Wallis test was performed with log normalized data to identify imbalances in abundance only. Significance level was p < 0.05.

### General information on statistical analysis

All downstream computations were performed in R v3.2.2. P-values in multiple testing scenarios were corrected by false discovery rate.

## Results

After a rigorous quality check and preprocessing, which removed about 20% of the sequences, all sequences were normalized to 7000 sequences per sample, resulting in a total of 187,807 OTUs with a median of 1970 OTUs per sample.

### ADHD status associated with decreased alpha diversity

**Fig 1:**
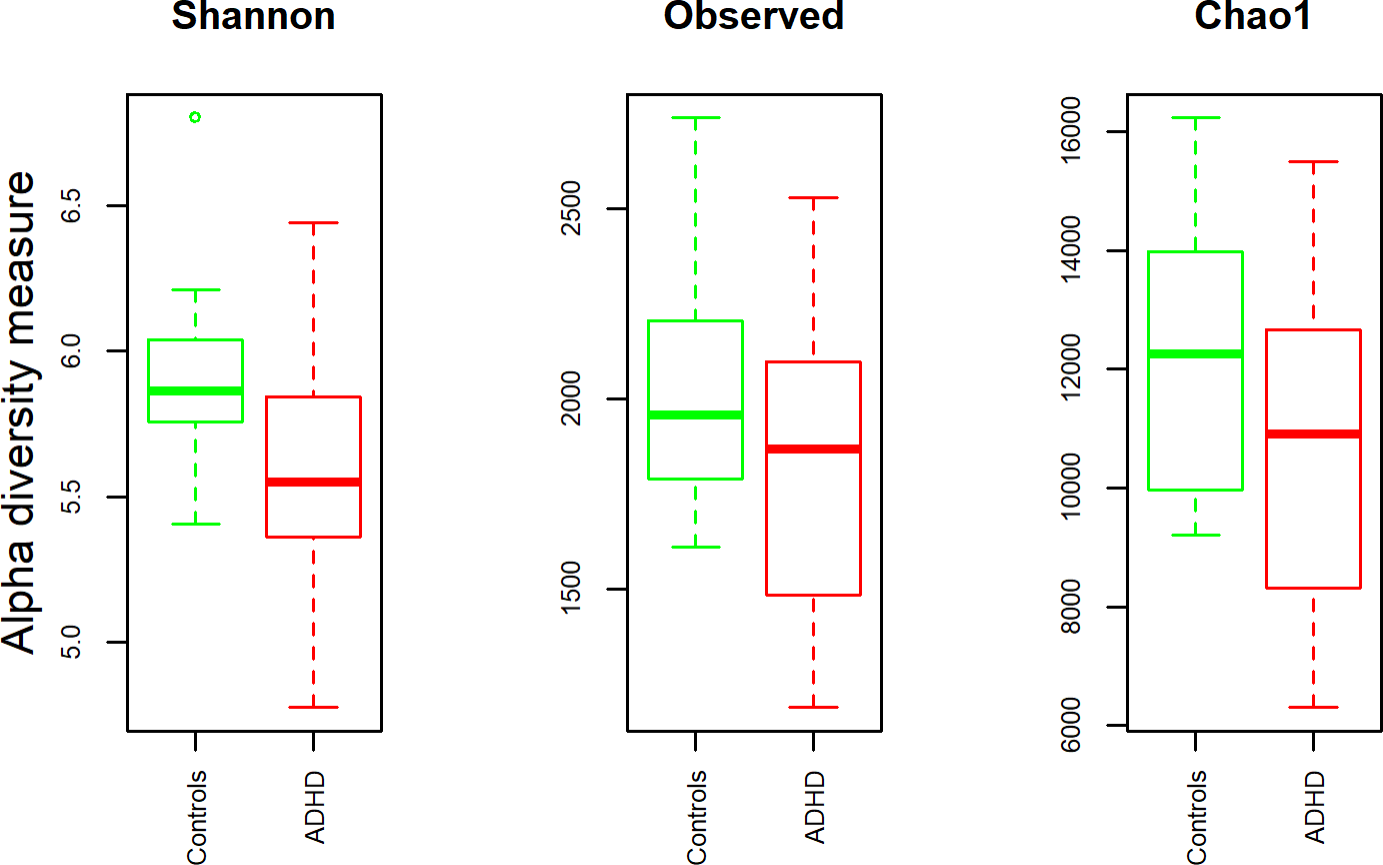
Alpha diversity of stool samples. Alpha-diversity, measured by observed species and Shannon diversity Index is plotted for patients with ADHD (red) and controls (green). The line inside the box represents the median, while the whiskers represent the lowest and highest values within the 1.5 interquartile range (IQR). Outliers as well as individual sample values are shown as dots. Statistical testing showed no difference for observed species (p_Observed_ = 0.25), while Shannon diversity was significantly decreased in ADHD compared to controls (p_Shannon_ = 0.036).

Alpha diversity was quantified by Shannon diversity index, which relates both OTU richness and evenness, and by the total number of observed species. Fig 1 shows the alpha diversity measurements for ADHD children versus controls. Statistical testing showed no difference for the observed species (p_Observed_ = 0.25) and Chao1 richness estimator (p_Chao1_ = 0.17), while Shannon diversity was significantly decreased in ADHD compared to controls (p_Shannon_ = 0.036). Regarding the parents, mothers of ADHD patients also showed a reduction in alpha diversity (p_Shannon_ = 0.029, p_Observed_ = 0.017), while fathers of ADHD patients and controls did not differ significantly (p > 0.05, see also S1 Fig and S1 Table). Four out of fourteen ADHD children did not receive ADHD medication. These patients showed a reduction in alpha diversity comparable to patients who suffered from ADHD but were treated with MPH (median_MPH_ = 5.53, median_no_MPH_ = 5.62, see S2 Fig).

### ADHD children and controls differ in microbial composition

**Fig 2:**
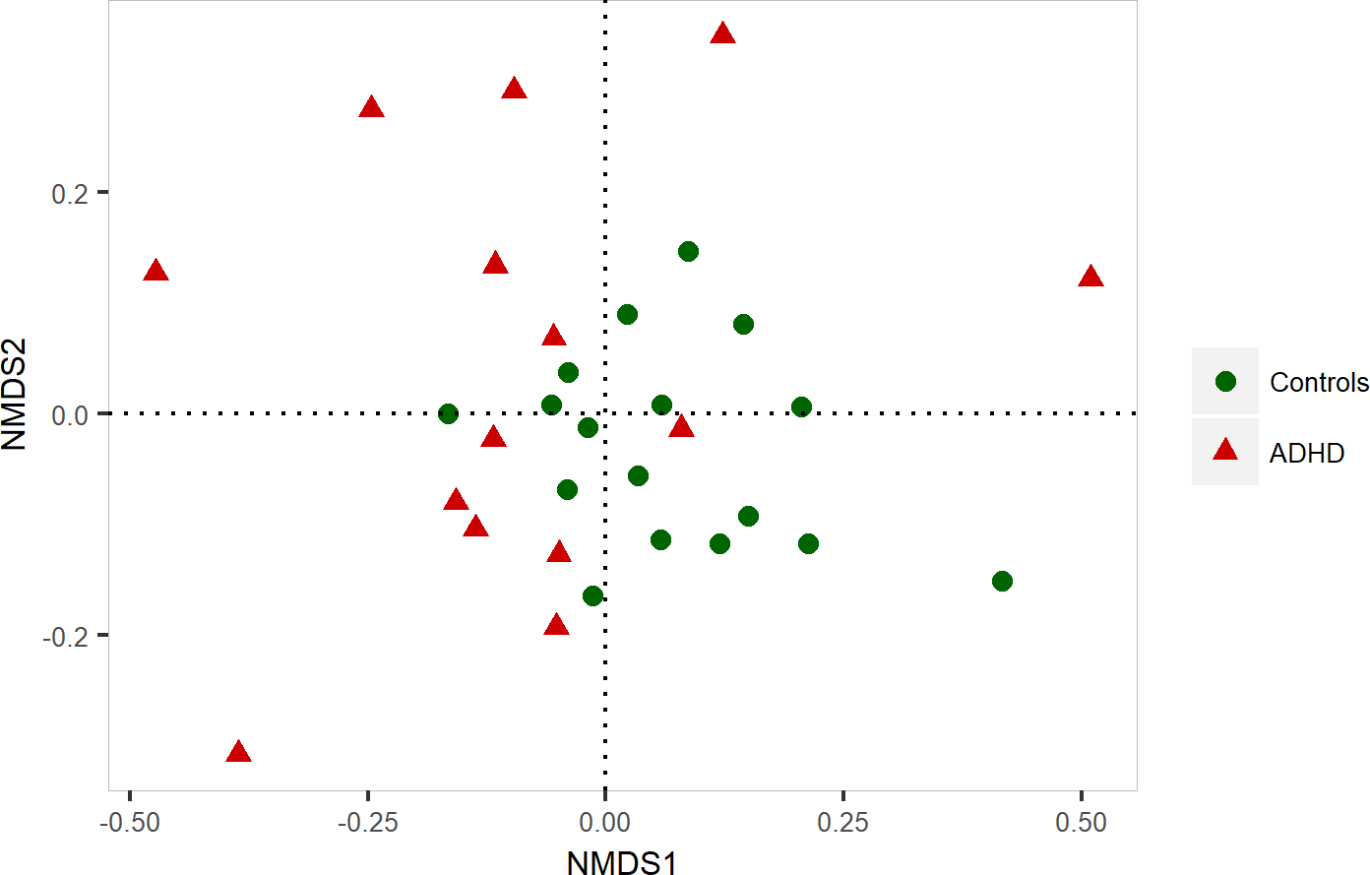
Non-metric multidimensional scaling (NMDS) of ADHD samples and healthy controls. NMDS is an unconstrained, distance-based ordination method which was performed with Bray-Curtis dissimilarity. Points represent samples. Samples that are more similar to one another are ordinated closer together. ADHD patients are plotted as red triangles, and controls are represented as green dots. The groups show significant differences in similarity tested by ANOSIM (p_ANOSIM_ = 0.033).

As rank-based approaches, NMDS and ANOSIM were applied in order to test for dissimilarities in the microbial composition between ADHD patients and controls. NMDS results are displayed in Fig 2. Patients with ADHD (red dots) showed a shift to the left, which indicates compositional differences, and is confirmed by a significant result in the ANOSIM (p_ANOSIM_ = 0.033). To get more precise information about the differences between the two sample groups, tests for similarity of composition (ADONIS) and homogeneity (betadisper) were performed. Both showed significant differences between ADHD children and controls (p_ADONIS_ = 0.006, p_betdisper_ = 0.002). Inter-personal variation patterns of different phylogenetic levels can be found in S3, S4, S5 and S6 Figs as well as a comparison of dominant taxa in S7, S8, S9 and S10 Figs. Mothers and fathers of ADHD patients and controls did not differ significantly in microbia composition (p > 0.05), while ADHD mothers differed significantly from the ADHD patients (p_ADONIS_ = 0.037) and control children (p_ADONIS_ = 0.005). A RDA plot comparing the family member can be found in S11 Fig.

### Specific OTUs

**Fig 3:**
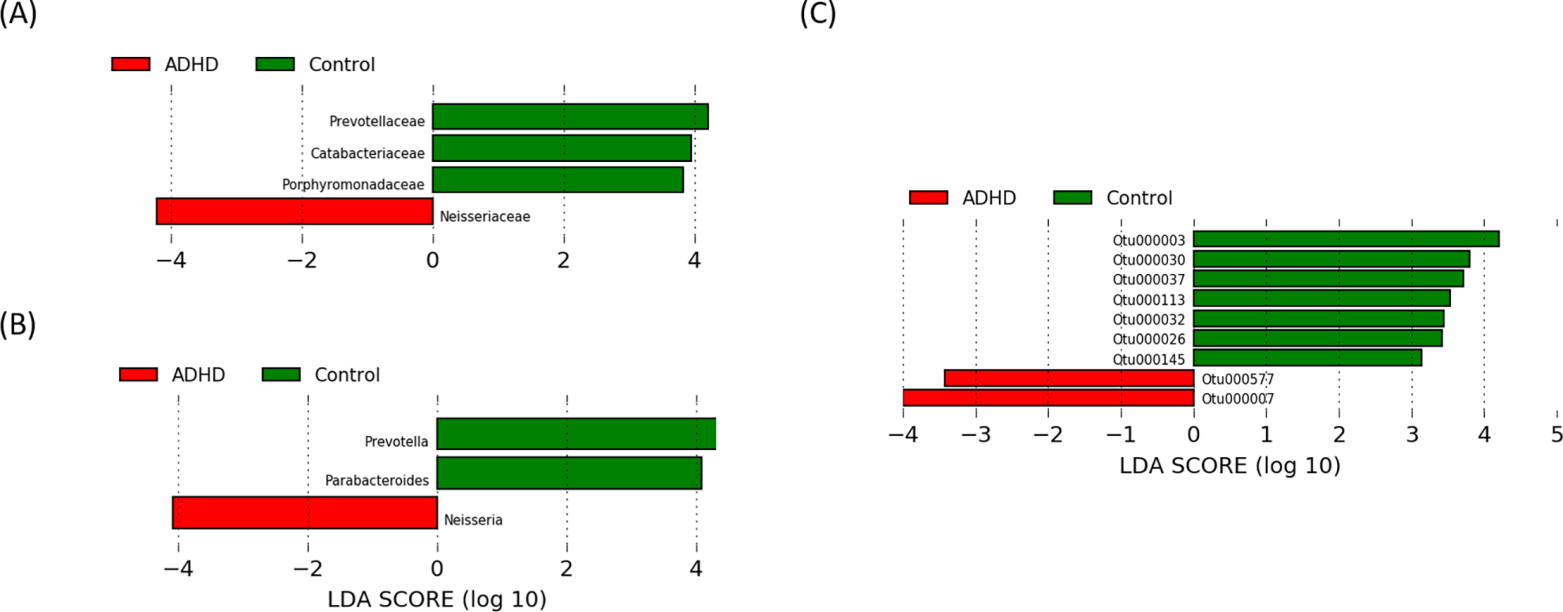
Results of LDA effect size (LefSe) analysis of male ADHD patients compared to healthy controls. The LEfSe analysis finds taxa which are significantly more abundant in one group, while the bar size represents the effect size of the taxa in the particular group. (A) family level, (B) genus level, (C) OTU level (97% similarity). There were no taxa differences at the order and phylum level. The threshold p-value was 0.05.

The LEfSe test for biomarkers was used in order to find significantly imbalanced OTUs, which showed the strongest effects for group differentiation. Analysis at the OTU level uncovered two ADHD-associated species belonging to the genera *Bacteroides* (OTU_7, OTU_577, Fig 3). At the genus level, *Prevotella* and *Parabacteroides* were detected as markers for the control group and *Neisseria* for the ADHD group. Analysis at the family level showed elevated levels of Prevotellaceae, Catabacteriaceae, and Porphyromonadaceae for healthy controls and Neisseriaceae for the ADHD children. At the phylum level no significant differences were observed. LEfSe takes into account both differences in abundances and frequency. Comparison of abundances by Kruskal-Wallis test revealed that, in contrast to the other Biomarker, the genus *Neisseria* did not differ in abundance, but only in frequency between the sample groups. Furthermore, the ADHD patients showed higher abundances in the family Bacteroidaceae (see also S8 Fig). Evaluation of the influence of the parental microbiome showed that the ADHD patients share slightly more OTUs with the father than with the mother (shared_father_ = 7,7%, shared_mother_ = 6,8%), while controls share equal amounts with fathers and mothers (shared_father_ = 5,9%, shared_mother_ = 5,8%). Abundances of possible biomarkers found by LEfSe analysis between patients and controls can be found for all family members in S12, S13 and S14 Figs.

### Associations between parental ratings and alpha diversity/beta diversity

**Fig 4:**
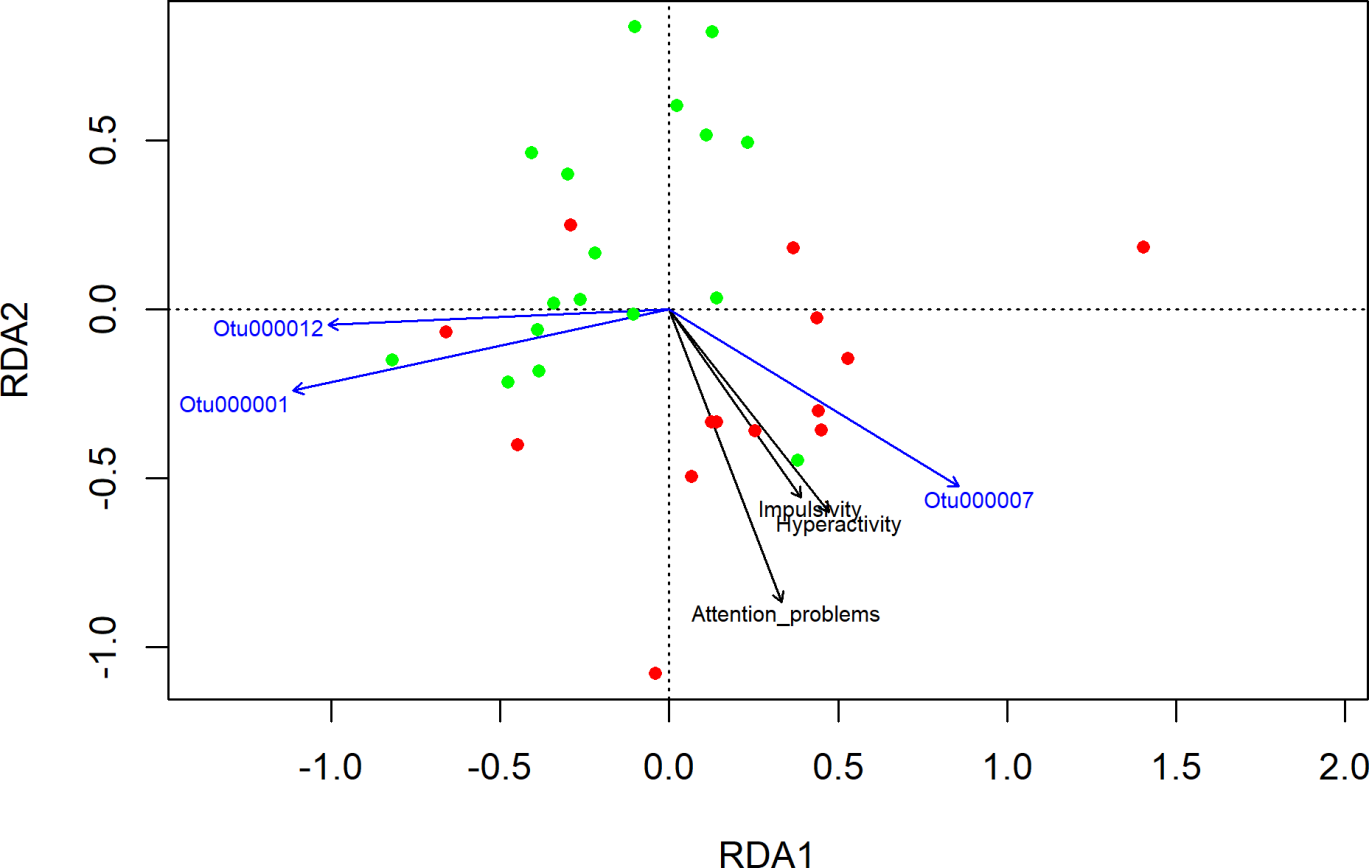
Differentiation of participants´ microbiomes. **RDA biplot at OTU level with Hellinger-transformed data.** Redundancy analysis is a constrained method based on multiple linear regression which enables correlation of explanatory variables with the RDA axes. The black dots represent individuals without ADHD; the green dots, individuals with ADHD diagnosis. Species and factors correlated with the RDA axes were determined by the envfit function of the R package vegan, the cut-off for plotted results was p = 0.01.

A linear model was used to determine associations in levels of attentional deficits, hyperactivity, and impulsivity (parental ratings as assessed by the FBB-HKS questionnaire) with mircobial alpha diversity. Levels of hyperactivity were significantly correlated with a change in alpha diversity (hyperactivity: r = -0.35, p = 0.03, impulsivity: r = -0.22, p = 0.13, attention problems: r = -0.15, p = 0.28). There were no significant correlations between the microbiome and clinical symptoms assessed by the CBCL questionnaire (for all CBCL scales r > 0.2, p > 0.2). Beta diversity and correlated species were examined by RDA on Hellinger transformed data (tb-RDA). An RDA analysis at the species level revealed that OTU_7 (*Bacteroides spec*.) correlated with levels of hyperactivity and impulsivity (Fig 4).

## Discussion

In this study, we observed that boys with ADHD have significantly reduced gut microbial diversity and show differences in microbial composition compared to healthy controls. We found that these differences are mainly caused by the family Prevotellaceae and Neisseriaceae. At the genus level, *Prevotella*, *Neisseria*, and two specific OTUs found as a potential biomarker for the ADHD. Furthermore, we found a negative correlation between symptoms of hyperactivity and alpha diversity.

These data are in line with the growing body of evidence for a bidirectional relationship between the gut microbiome and mental health [64–67]. Similarly, human studies lead to the conclusion that the microbiome is involved in psychopathology, such as in autism, depression, anxiety, obesity, or anorexia nervosa [30–34, 42, 43]. We found *Neisseria* and *Bacteroides spec*. as possible ADHD-associated biomarkers. Both genera contain commensal species which are part of the healthy human microflora [68, 69]. Although our method did not allow the determination of the particular species within *Neisseria* and *Bacteroides*, there are well-known pathogens in these genera that might be involved in ADHD pathogenesis. Assuming a causal role in ADHD, especially the brain-invading capability of *N*. *meningitides* might be of interest. Berg and colleagues found that post-meningitic children showed significantly more symptoms in the areas of inattention, hyperactivity and impulsiveness than their siblings [70]. *N*. *gonorrhoeae*, on the other hand, uses host-derived sialic acid for its lipopolysaccharides as a mechanism to evade immune defense [71]. This is linked to ADHD by a genome-wide analysis that found an lncRNA gene ENST00000427806 associated with aggressiveness in ADHD [72]. The target gene for this lncRNA is a protein‐coding a sialyltransferase gene (ST6GALNAC5). Thus, changes in sialic acid metabolism in ADHD could be used by *Neisseria* to escape the host immune defense, which might explain the observed overrepresentation of this genus in our ADHD samples.

The LEfSe analysis results showed that the family Prevotellaceae was significantly more abundant in controls, while the ADHD patients showed elevated levels of Bacteroidaceae. Bacteroidetes generate essential vitamins and cofactors, and processing constituents such as fiber, making them beneficial in support of human immunity, physiology, biochemistry, and neurochemistry [73, 74]. The LEfSe analysis revealed two *Bacteroides* species (OTU_7 and OTU_577) as potential biomarkers for the ADHD group. Members of the genus *Bacteroides* are usually beneficial for the gut microbiota, but they are also capable of producing extraordinarly complex mixtures of amyloids, lipopolysaccharides, enterotoxins and neurotoxins, which can affect the blood brain barrier´s structure as well as the central nervous system [75].

Assuming a causal relationship, the reduced alpha diversity that we found in ADHD patients might reflect a bacterial community involved in deviant neural transmission. Many bacterial species are able to produce GABA [76], which is the main inhibitory neurotransmitter in the human cerebral cortex. GABA is antiproportionally correlated with impulsivity, and GABA levels are reduced in young patients with ADHD [77]. Other studies have shown that the gut microbiota affects levels of excitatory and inhibitory neurotransmitters (i.e. serotonin, GABA, and dopamine), while germ-free mice tend to have lower levels of neurotransmitter precursors like tryptophan, tyrosine, and glutamine in the brain compared to re-colonized mice [78, 79]. Possibly in line with this, we found a negative correlation between the hyperactivity score and the alpha diversity. This confirms findings from a mouse model of germ-free mice that not only displayed an altered stress response but also an increased level of motor activity compared to conspecifics with a normal, functional microbiota [44]. A reconstitution of the microbiota reversed alterations in both stress response and motor activity. In the light of these facts, our results lead to the assumption that the impact of the microbiome on hyperactivity is more pronounced than the impact on attention deficits. This dissociation would also explain the lack of a correlation between the alpha diversity and ratings of global ADHD symptomatology as assessed by the CBCL.

By including parents in the analyses, we also observed that mothers of ADHD patients compared to mothers of healthy controls showed a reduced alpha diversity. Accordingly, ADHD self-ratings revealed that mothers of patients displayed more ADHD symptoms in the past and present than mothers of healthy controls did. There were no differences in alpha diversity and self-ratings between the fathers of patients and controls. With a heritability of about 76%, ADHD is a familial disorder, and its relative risk is about 5–9 in first-degree relatives [12, 13, 80]. Although males are more often affected than females (estimated ratio 3–4:1) [80], our alpha-diversity data suggest that alterations in the microbiome composition might be passed on maternally. Actually, patients share more OTUs with the father than with the mother, while mothers and patients differ significantly in microbial composition (beta-diversity). This would argue against maternally heredity of ADHD microbiota. However, a comparison of adult mothers’ microbiota with juvenile ADHD patients might be inadequate, considering that adult and juvenile, as well as female and male [81, 82], microbiota differ per se. Nevertheless, an influence of maternal microbiota during a critical developmental window or influence by inherited genotypes cannot be excluded. However, we have no comprehensive information about parents´ mental health (no diagnostic interview was made, no clinical confirmation of ADHD in the past or present, and no information about possible comorbidities or medication was obtained). Therefore, we had to refrain from interpreting these results here. However, we suggest that parents should be included in the diagnostic process in future studies. In addition, longitudinal studies would be needed to elucidate the critical time window of the maternal influence on the patients’ microbiota.

Patients and controls did not differ with respect to BMI and the reported intake of meat, fruits/vegetables, yoghurt, other milk products, or fast food during the month prior to stool donation. All these foods are well-known to influence the intestinal microbiota [83–85]. Therefore, group differences in alpha diversity may be an indication of a biomarker for ADHD and not the result of group-specific nutrition. However, our study may also provide the basis for a supportive treatment strategy in ADHD, since the microbiome is influenced by nutrition [26, 86]: it has already been shown that food constituents significantly interact with the ADHD symptom burden [7, 10, 11, 14, 15, 17–22], while supplementation of free fatty acids, as well as the exclusion of artificial food colors or other additives can attenuate ADHD symptom load [87, 88]. Moreover, one study suggested that the supplementation of probiotics in younger ages reduces the risk for neurodevelopmental disorders (including ADHD) [45]. Although the underlying mechanisms of a diet-associated severity of ADHD symptoms are not understood, the microbiome has been suspected as being the missing link [37, 89].

A limitation of the study is the concomitant medication: Ten of 14 patients had taken methylphenidate (MPH), the first-line treatment of ADHD, for more than one year. Nine of them discontinued taking the medication at least 48h (approximately twelve half-lives) prior to sample collection. MPH increases the availability of dopamine by blocking the dopamine transporter in the CNS [90–92], reducing symptoms of inattention and hyperactivity [93–95]. To date, no information is available as to whether or not MPH affects the bacterial composition in the gut. Therefore, we cannot exclude that MPH (at all or even after more than 48h of washout time) had an impact on gut bacteria. MPH has chemical similarities to cocaine [96] with comparable effects on the dopamine transporter [97–99]. One animal study suggested that the experimental reduction of gut microbiome, as induced by antibiotics, predicts the host´s response to stimulants such as cocaine: the lower the bacteria level, the higher the behavioral abuse response [100]. One human study revealed that the acute intake of cocaine leads to a higher relative abundance of Bacteroidetes compared to non-users, but there were no differences in alpha diversity between groups [101]. If MPH had an influence on microbial alpha diversity in ADHD after more than 48h of washout time, then the results of the abovementioned study indicate that this would more likely have resulted in an underestimation of the reduction in alpha diversity caused by MPH. Our limited data on this issue (n = 4 with ADHD but no medication) indicate that the alpha diversity in young ADHD patients is at least not substantially affected by medication intake. Thus, further studies are required to unravel a possible yet unknown effect of MPH on the microbiome in ADHD.

Another limitation is the small sample size of 14 patients and 17 controls. Studies with larger cohorts are required not only to replicate our findings in a medication-controlled sample but also to investigate possible differences in alpha diversity between subtypes of ADHD. Moreover, including females is mandatory to investigate possible gender effects as indicated by the parental microbiome. In addition, future studies can be designed to develop effective dietary guidelines or treatment strategies with beneficial bacterial species (probiotics) [45] or specific nutritional components for the prevention and treatment of ADHD [37, 87, 102, 103]. Finally, longitudinal studies are needed to further unravel the precise differences between healthy and ADHD-affected children with regards to the gut microbiome over the course of disease development.

Taking the small sample size and the concomitant medication into account, our findings support the hypothesis of an ADHD-specific microbiota. We suggest that the genus *Neisseria* and elevated levels of *Bacteroides spec*. are associated with juvenile ADHD.

## Supporting information

S1 Fig Comparison of parental alpha diversity. Alpha-diversity, measured by observed species (A) and Shannon diversity Index (B) is plotted for parents from ADHD patients (red) and parents from controls (green); IP, index patients; control, healthy controls. The line inside the box represents the median, while the whiskers represent the lowest and highest values within 1.5 interquartile range (IQR). Outliers as well as individual sample values are shown as dots. Statistical testing showed a difference in alpha diversity for mothers from ADHD patients and control mothers in observed species (p_Observed_ = 0.017) and Shannon diversity (p_Observed_ = 0.029), while fathers show no significant difference.
(JPEG)

S2 Fig Comparison of alpha diversity regarding medication. Alpha-diversity, measured by observed species (A) and Shannon diversity Index (B) is plotted for ADHD patients (red; MPH: patients on MPH treatment; n = 10; no_MPH: patients without medication; n = 4) and controls (green); IP, index patients; control, healthy controls. The line inside the box represents the median, while the whiskers represent the lowest and highest values within 1.5 interquartile range (IQR). Outliers as well as individual sample values are shown as dots. No significant differences were found.
(JPEG)

S3 Fig Phylum taxonomy distribution. Bar Plot showing the relative proportion of the bacterial phyla within all participants; P, patients; C, healthy controls.
(JPEG)

S4 Fig Family taxonomy distribution. Bar Plot showing the relative proportion of the top 20 bacterial families within all participants; P, patients; C, healthy controls.
(JPEG)

S5 Fig Genus taxonomy distribution. Bar Plot showing the relative proportion of the top 20 bacterial genera within all participants; P, patients; C, healthy controls.
(JPEG)

S6 Fig OTU taxonomy distribution. Bar Plot showing the relative proportion of the top 20 bacterial OTU within all participants; P, patients; C, healthy controls.
(JPEG)

S7 Fig Comparisons of bacterial phylum abundance. Box plot showing the abundances of bacterial phyla stratified by group (ADHD vs. controls).
(JPEG)

S8 Fig Comparisons of bacterial family abundance. Box plot showing the abundances of bacterial families stratified by group (ADHD vs. controls).
(JPEG)

S9 Fig Comparisons of bacterial genus abundance. Box plot showing the abundances of bacterial genera stratified by group (ADHD vs. controls).
(JPEG)

S10 Fig Comparisons of bacterial OUT abundance. Box plot showing the abundances of bacterial OTU stratified by group (ADHD vs. controls).
(JPEG)

S11 Fig Clustering of family members. Beta diversity as Hellinger-transformed redundancy analysis of ADHD samples versus healthy controls. The axes show the first three constrained axes from redundancy analysis (RDA1, RDA2, RDA3).
(PNG)

S12 Fig Abundance of selected bacterial families. Boxplot showing the abundance of significant bacterial families found by LEfSe analysis for participants and their parent; IP, index patients; control, healthy controls.
(JPEG)

S13 Fig Abundance of selected bacterial genera. Boxplot showing the abundance of significant bacterial genera found by LEfSe analysis participants and their parents; IP, index patients; control, healthy controls.
(JPEG)

S14 Fig Abundance of selected bacterial OTUs. Boxplot showing the abundance of significant bacterial OTUs found by LEfSe analysis for participants and their parents; IP, index patients; control, healthy controls.
(JPEG)

S1 Table Comparison of parental alpha diversity using pairwise Wilcoxon rank-sum tests. (DOCX)

S2 Table Comparison of alpha diversity regarding medication using pairwise Wilcoxon rank-sum tests. (DOCX)
