# Supplementary figures and images for: Reduced microbiome alpha diversity in young patients with ADHD
Source: PLoS One. 2018 Jul 12;13(7):e0200728. doi: 10.1371/journal.pone.0200728 (PMC6042771; doi:10.1371/journal.pone.0200728)

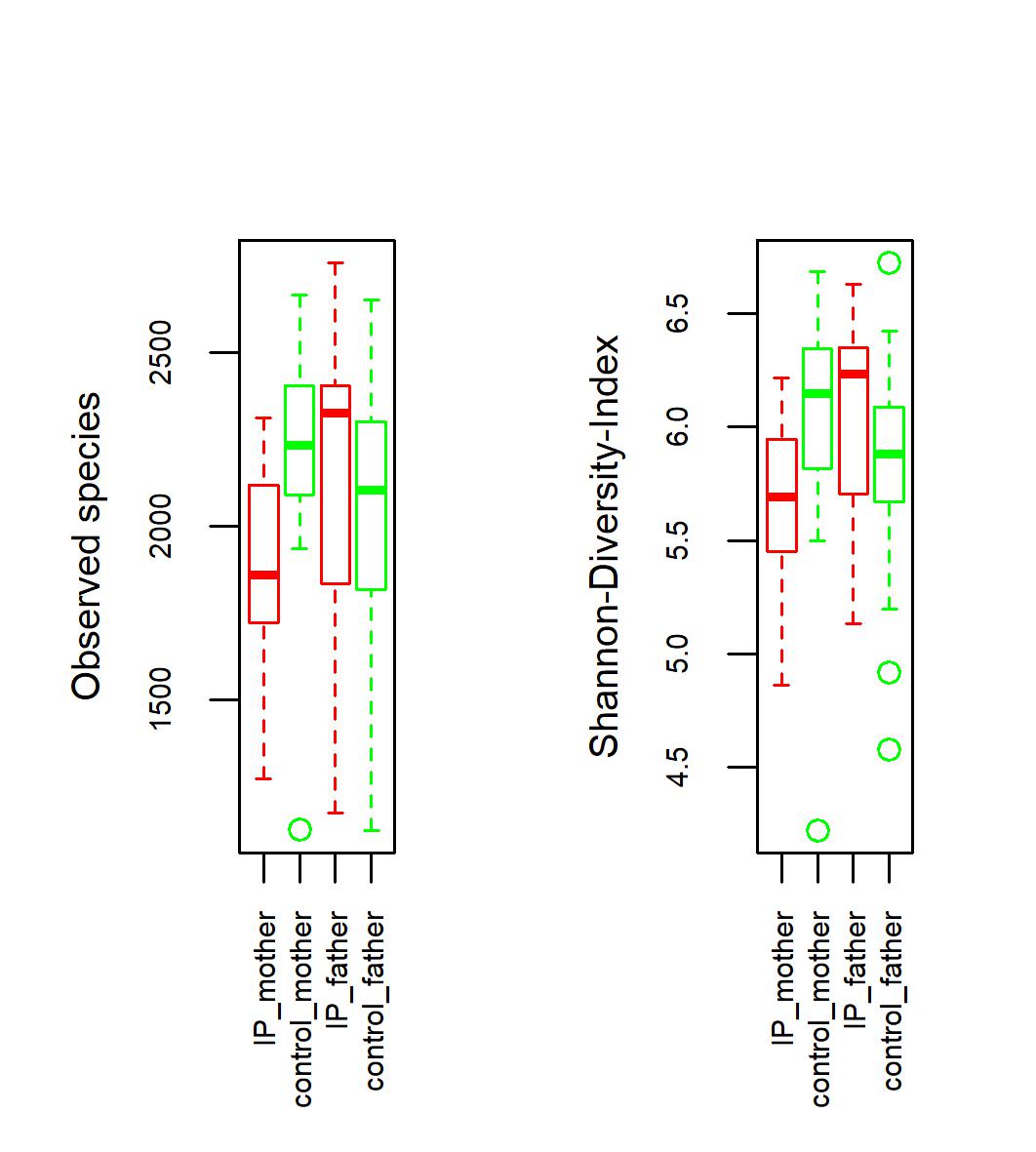

Supplement: S1 Fig — Alpha-diversity, measured by observed species (A) and Shannon diversity Index (B) is plotted for parents from ADHD patients (red) and parents from controls (green); IP, index patients; control, healthy controls. The line inside the box represents the median, while the whiskers represent the lowest and highest values within 1.5 interquartile range (IQR). Outliers as well as individual sample values are shown as dots. Statistical testing showed a difference in alpha diversity for mothers from ADHD patients and control mothers in observed species (pObserved = 0.017) and Shannon diversity (pObserved = 0.029), while fathers show no significant difference. (JPEG) [file pone.0200728.s001.jpeg]

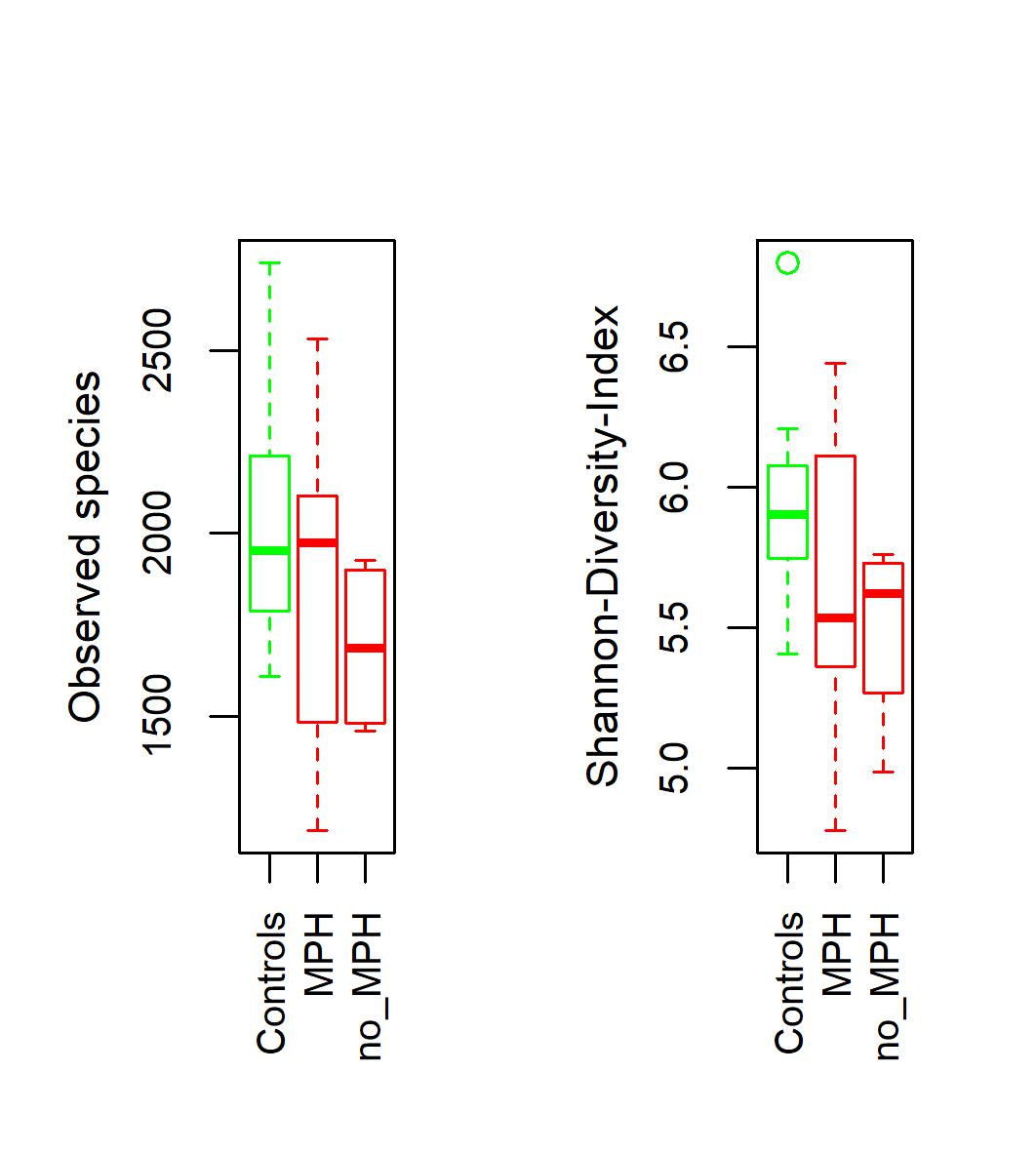

Supplement: S2 Fig — Alpha-diversity, measured by observed species (A) and Shannon diversity Index (B) is plotted for ADHD patients (red; MPH: patients on MPH treatment; n = 10; no_MPH: patients without medication; n = 4) and controls (green); IP, index patients; control, healthy controls. The line inside the box represents the median, while the whiskers represent the lowest and highest values within 1.5 interquartile range (IQR). Outliers as well as individual sample values are shown as dots. No significant differences were found. (JPEG) [file pone.0200728.s002.jpeg]

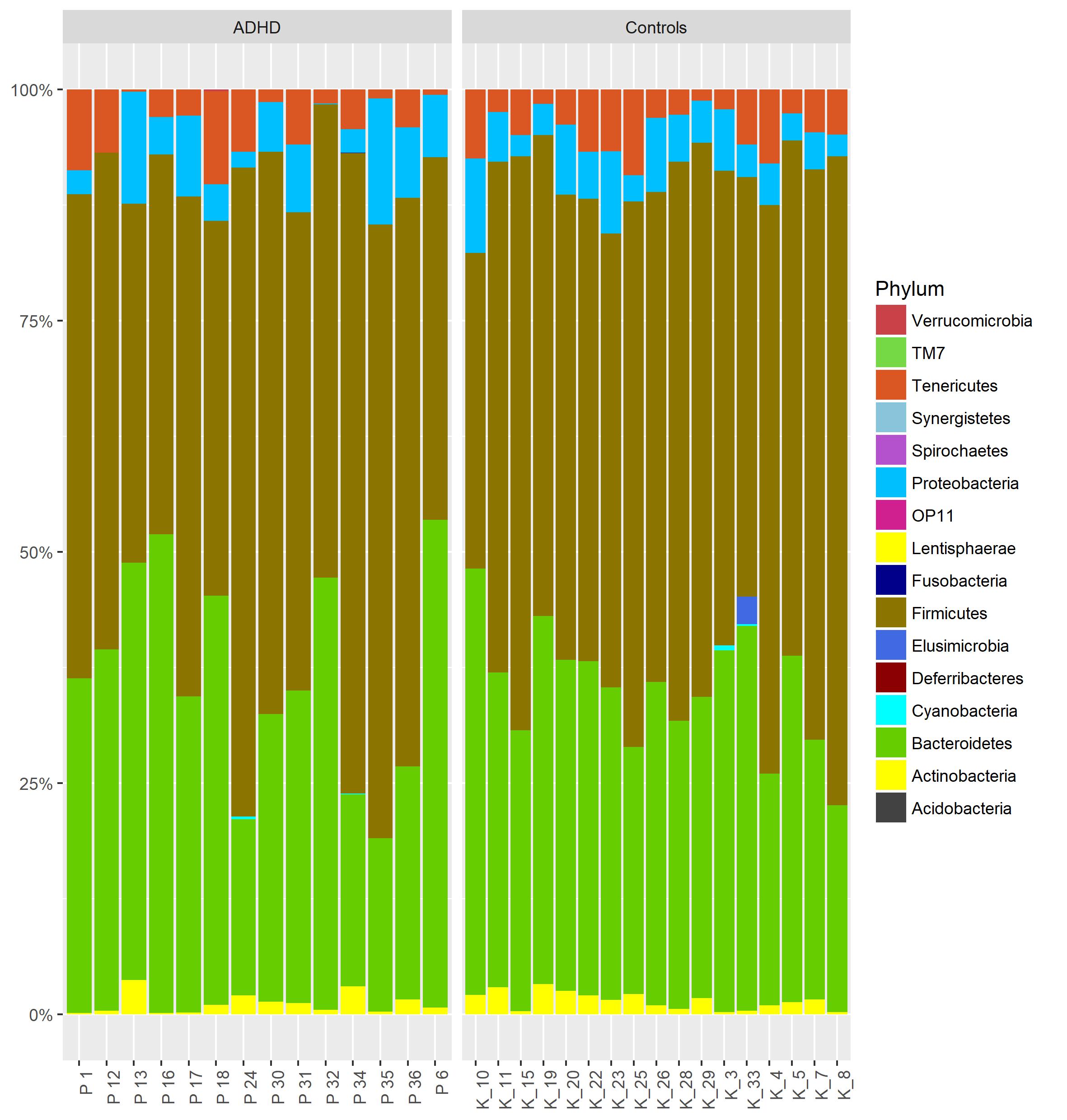

Supplement: S3 Fig — Bar Plot showing the relative proportion of the bacterial phyla within all participants; P, patients; C, healthy controls. (JPEG) [file pone.0200728.s003.jpeg]

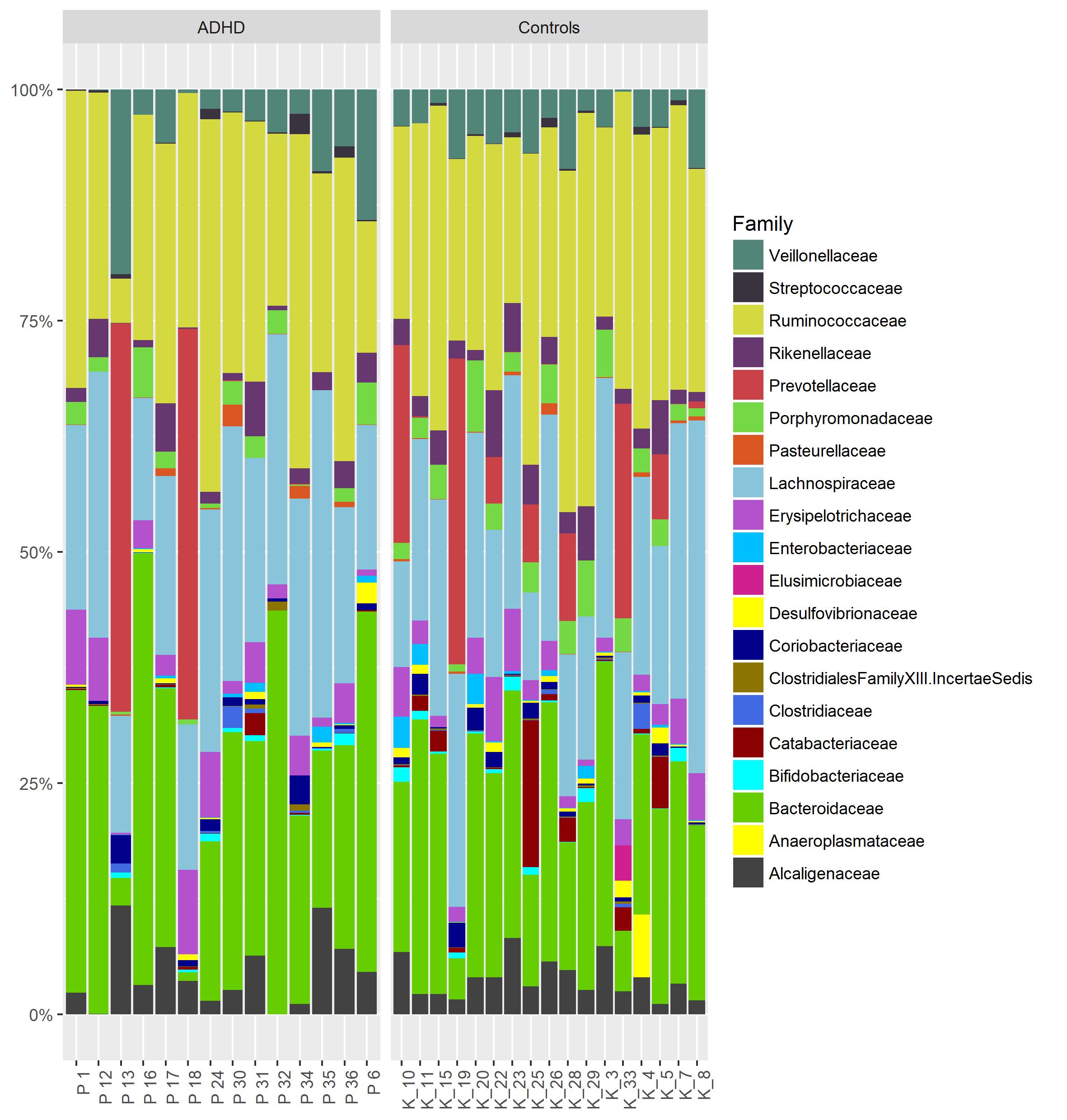

Supplement: S4 Fig — Bar Plot showing the relative proportion of the top 20 bacterial families within all participants; P, patients; C, healthy controls. (JPEG) [file pone.0200728.s004.jpeg]

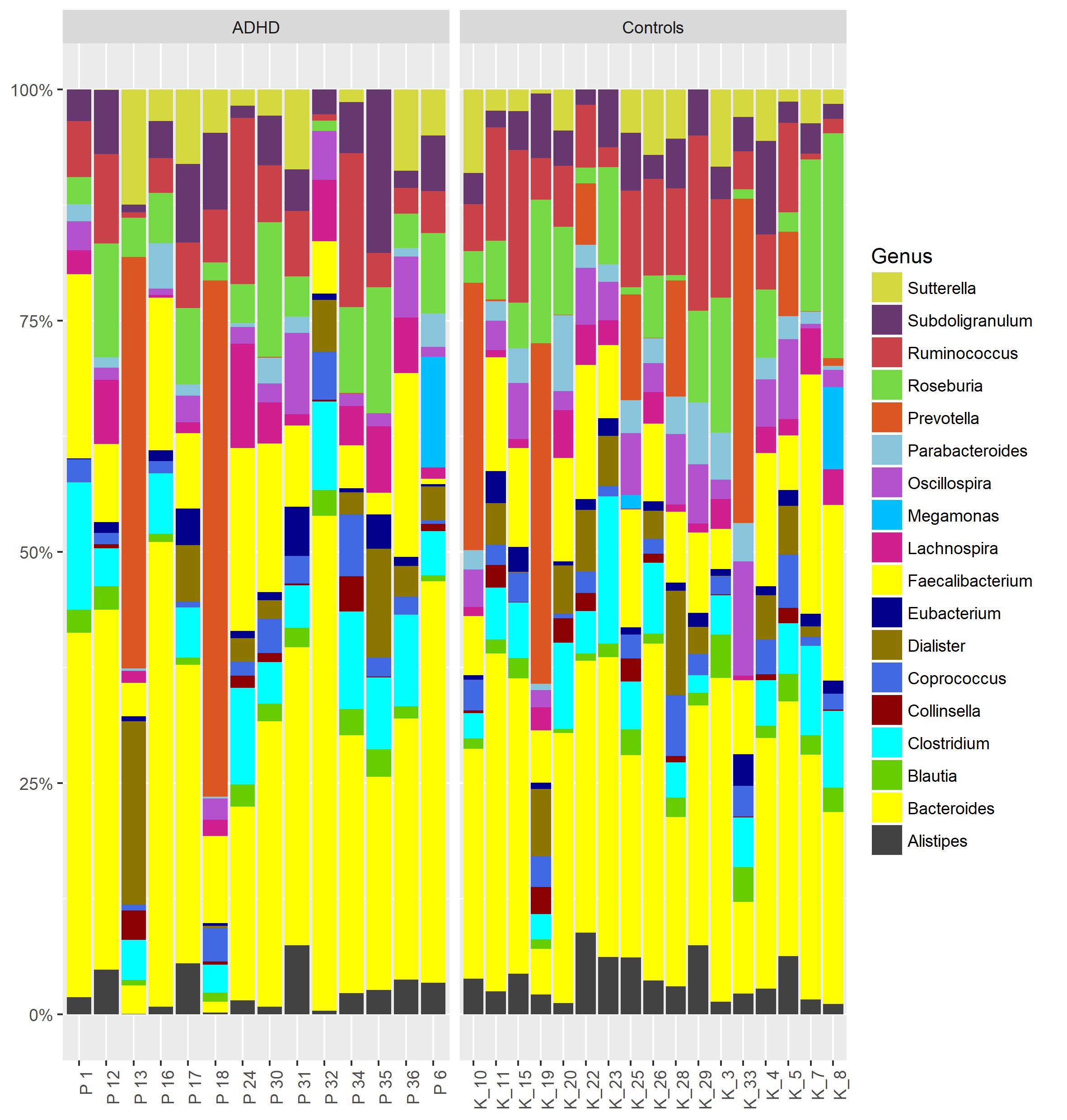

Supplement: S5 Fig — Bar Plot showing the relative proportion of the top 20 bacterial genera within all participants; P, patients; C, healthy controls. (JPEG) [file pone.0200728.s005.jpeg]

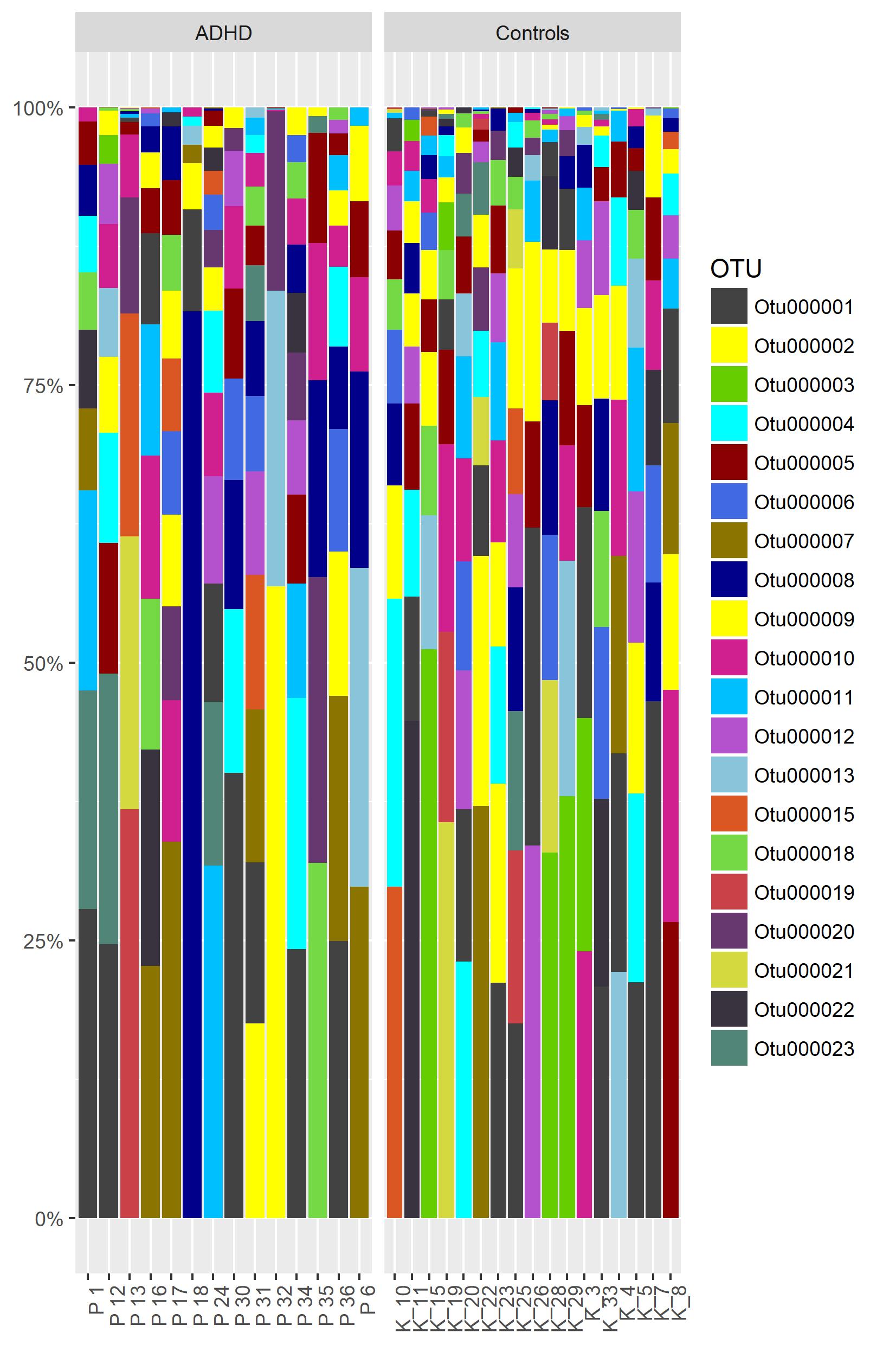

Supplement: S6 Fig — Bar Plot showing the relative proportion of the top 20 bacterial OTU within all participants; P, patients; C, healthy controls. (JPEG) [file pone.0200728.s006.jpeg]

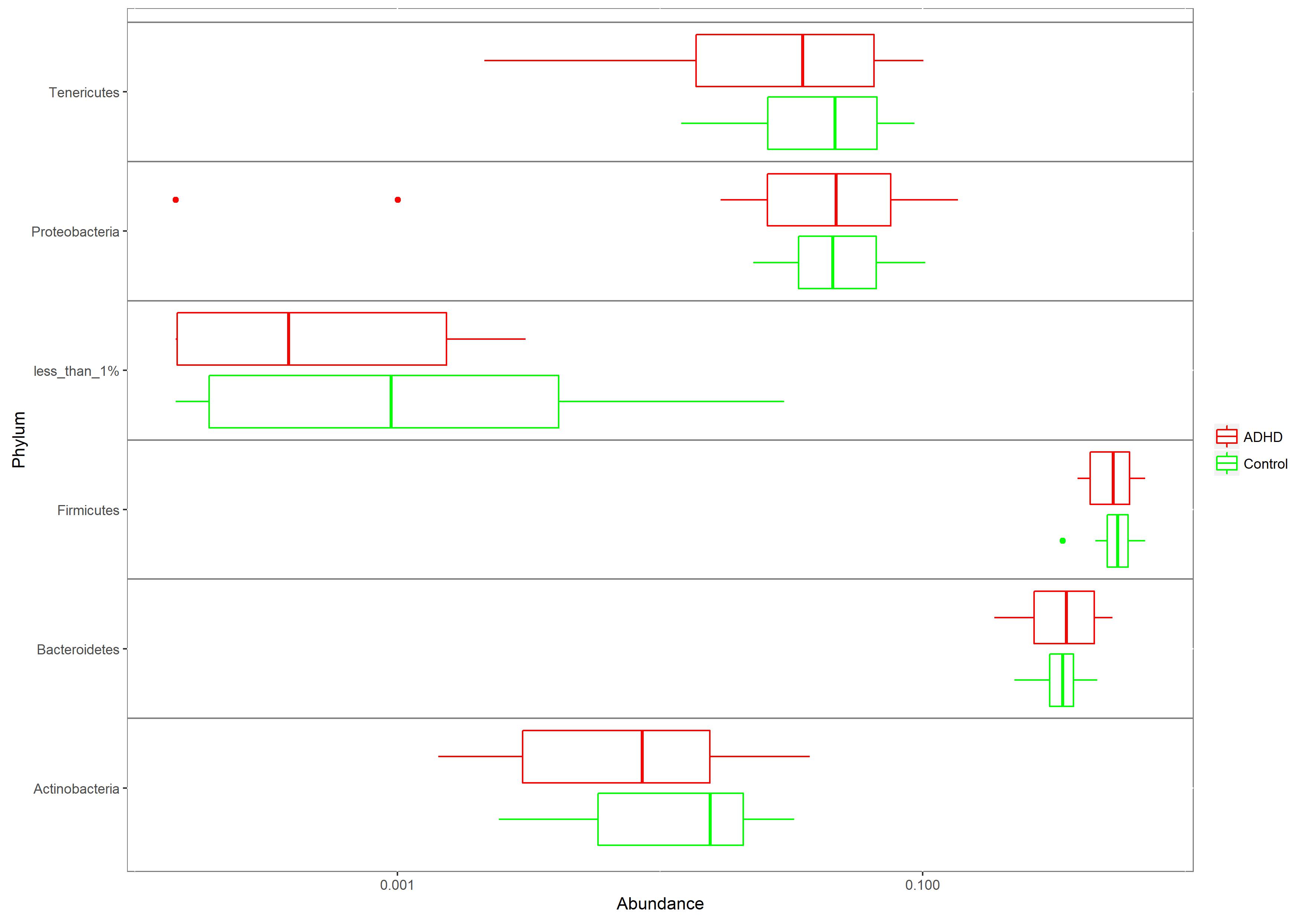

Supplement: S7 Fig — Box plot showing the abundances of bacterial phyla stratified by group (ADHD vs. controls). (JPEG) [file pone.0200728.s007.jpeg]

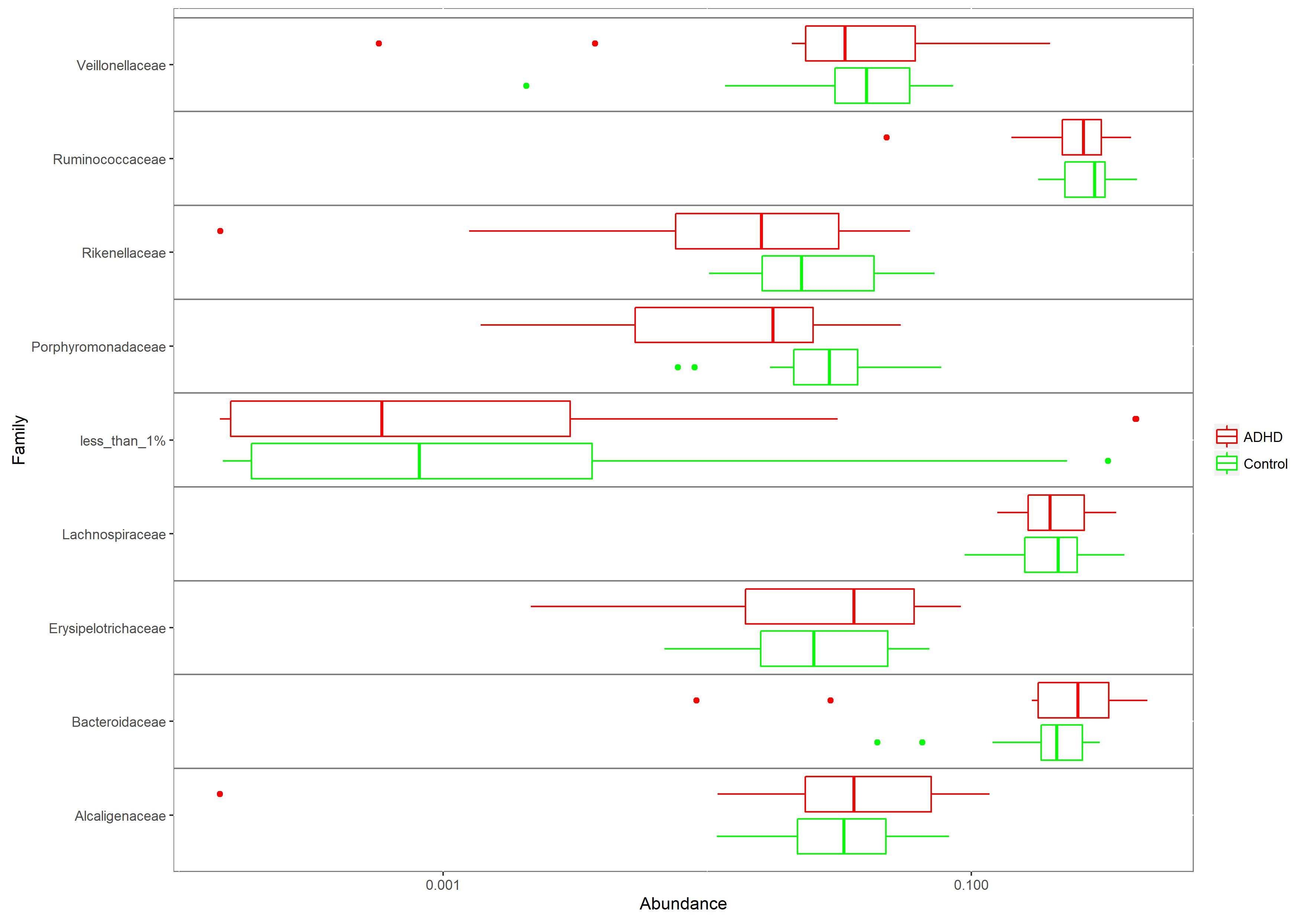

Supplement: S8 Fig — Box plot showing the abundances of bacterial families stratified by group (ADHD vs. controls). (JPEG) [file pone.0200728.s008.jpeg]

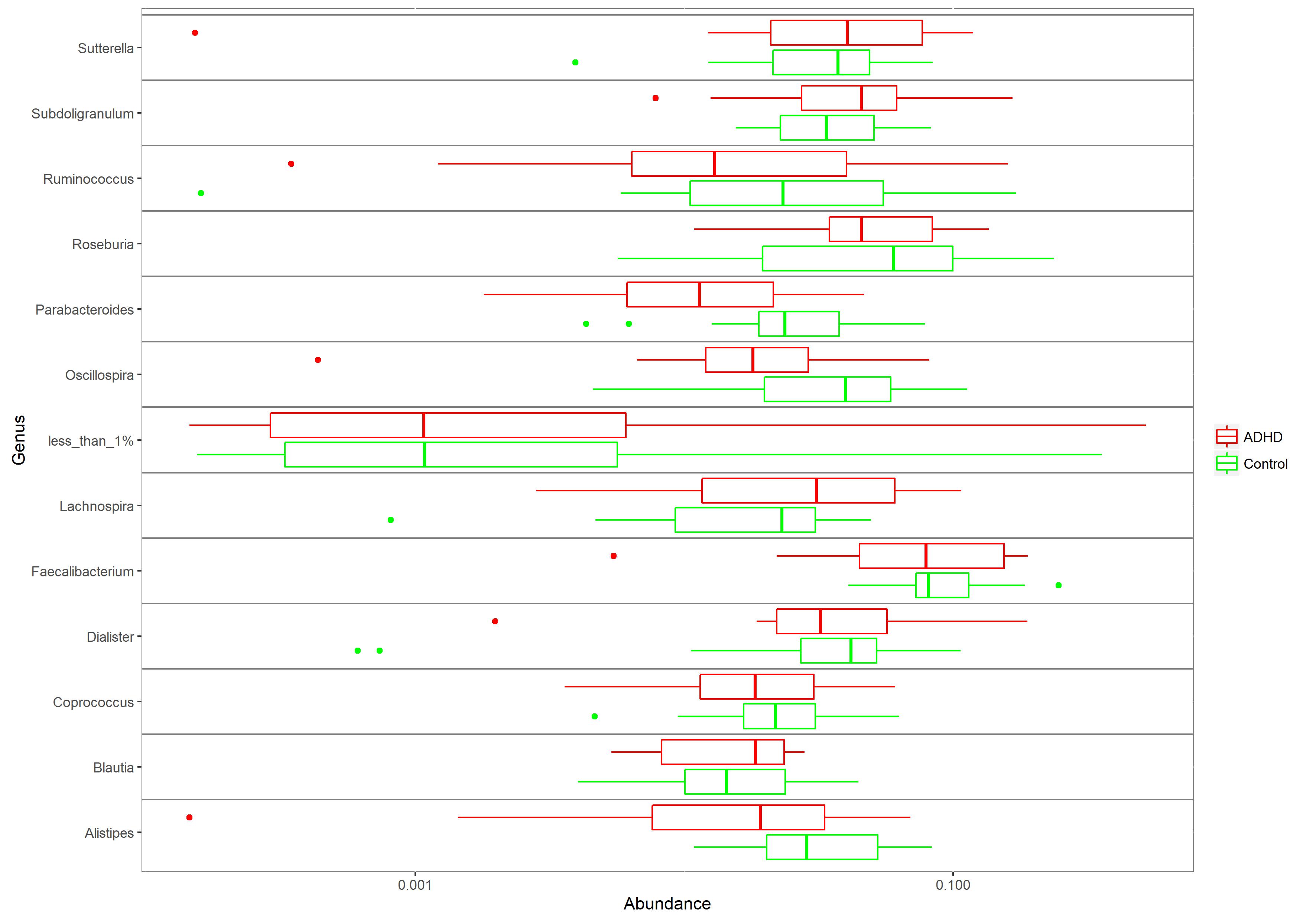

Supplement: S9 Fig — Box plot showing the abundances of bacterial genera stratified by group (ADHD vs. controls). (JPEG) [file pone.0200728.s009.jpeg]

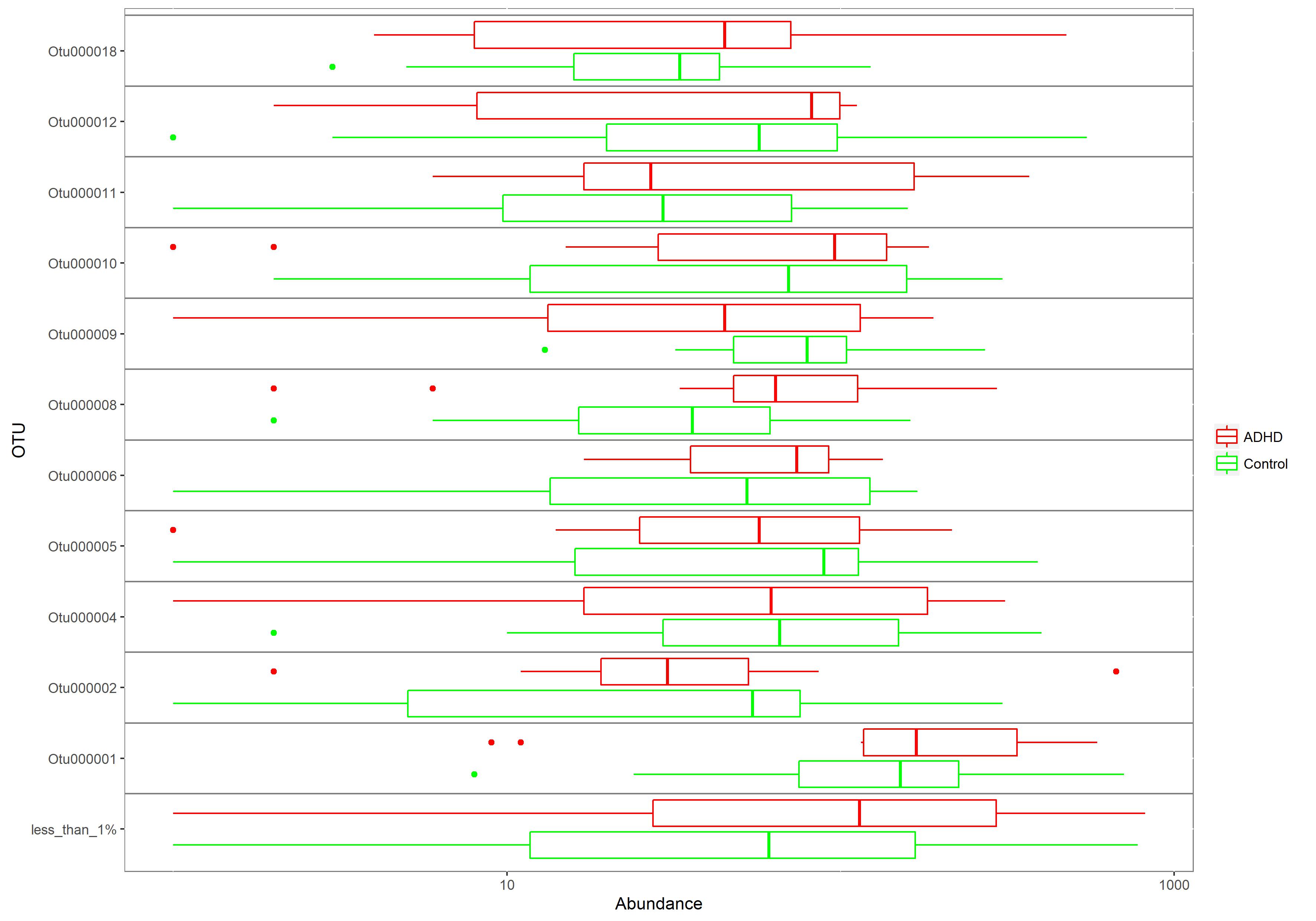

Supplement: S10 Fig — Box plot showing the abundances of bacterial OTU stratified by group (ADHD vs. controls). (JPEG) [file pone.0200728.s010.jpeg]

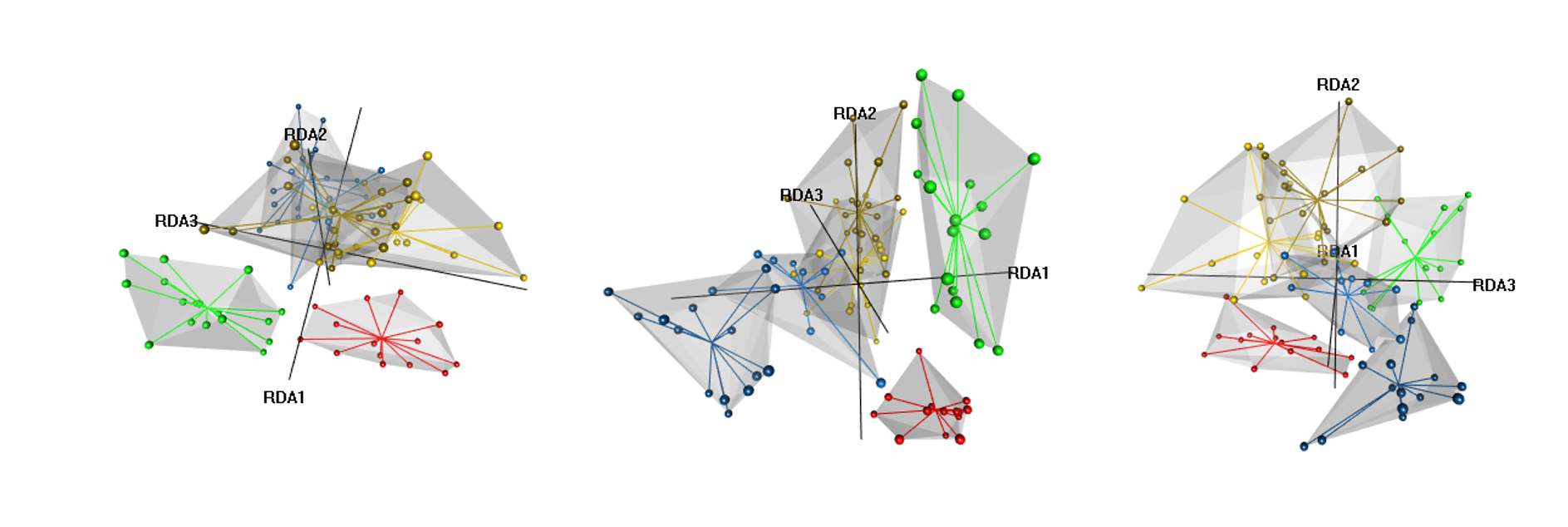

Supplement: S11 Fig — Beta diversity as Hellinger-transformed redundancy analysis of ADHD samples versus healthy controls. The axes show the first three constrained axes from redundancy analysis (RDA1, RDA2, RDA3). (PNG) [file pone.0200728.s011.png]

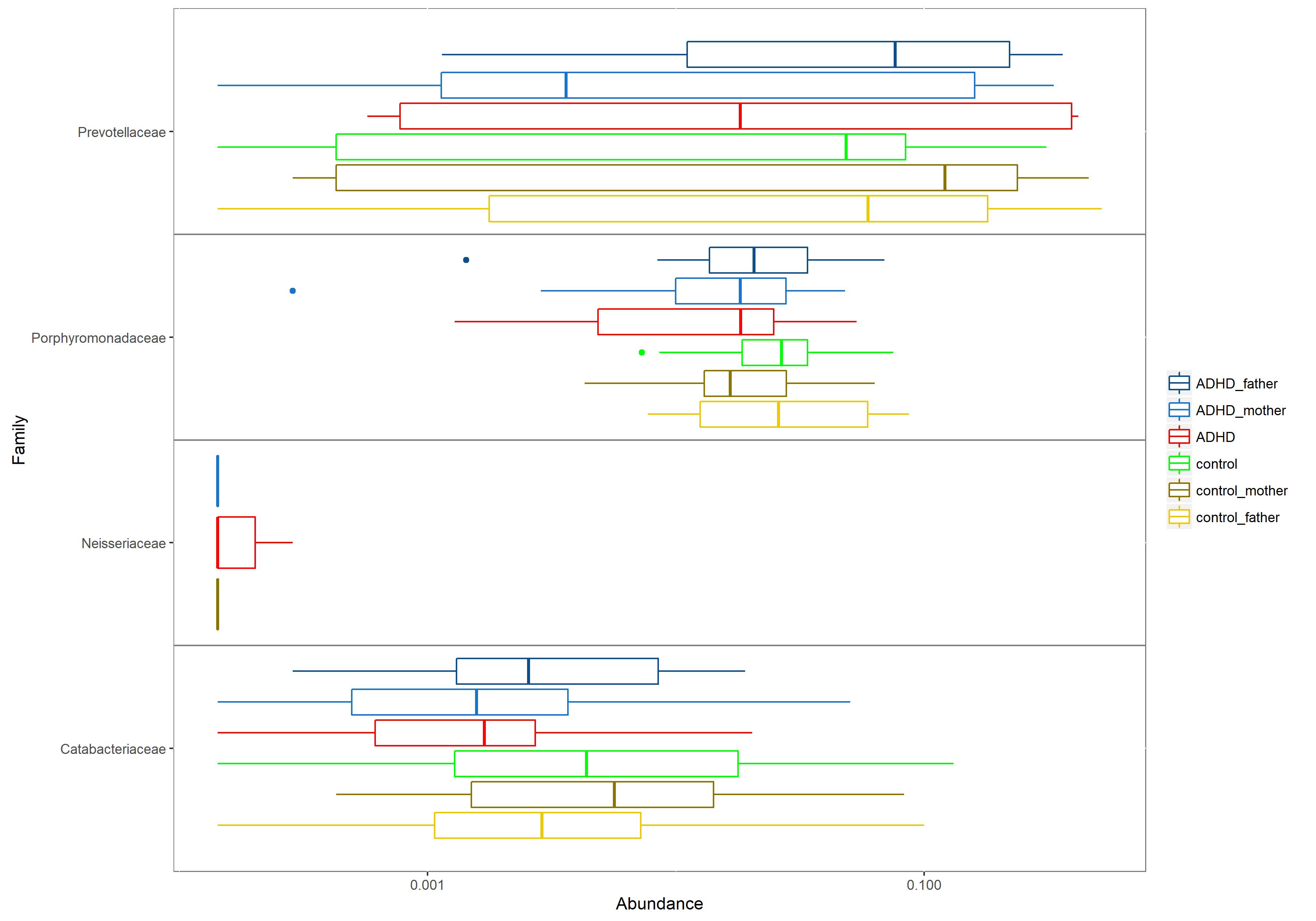

Supplement: S12 Fig — Boxplot showing the abundance of significant bacterial families found by LEfSe analysis for participants and their parent; IP, index patients; control, healthy controls. (JPEG) [file pone.0200728.s012.jpeg]

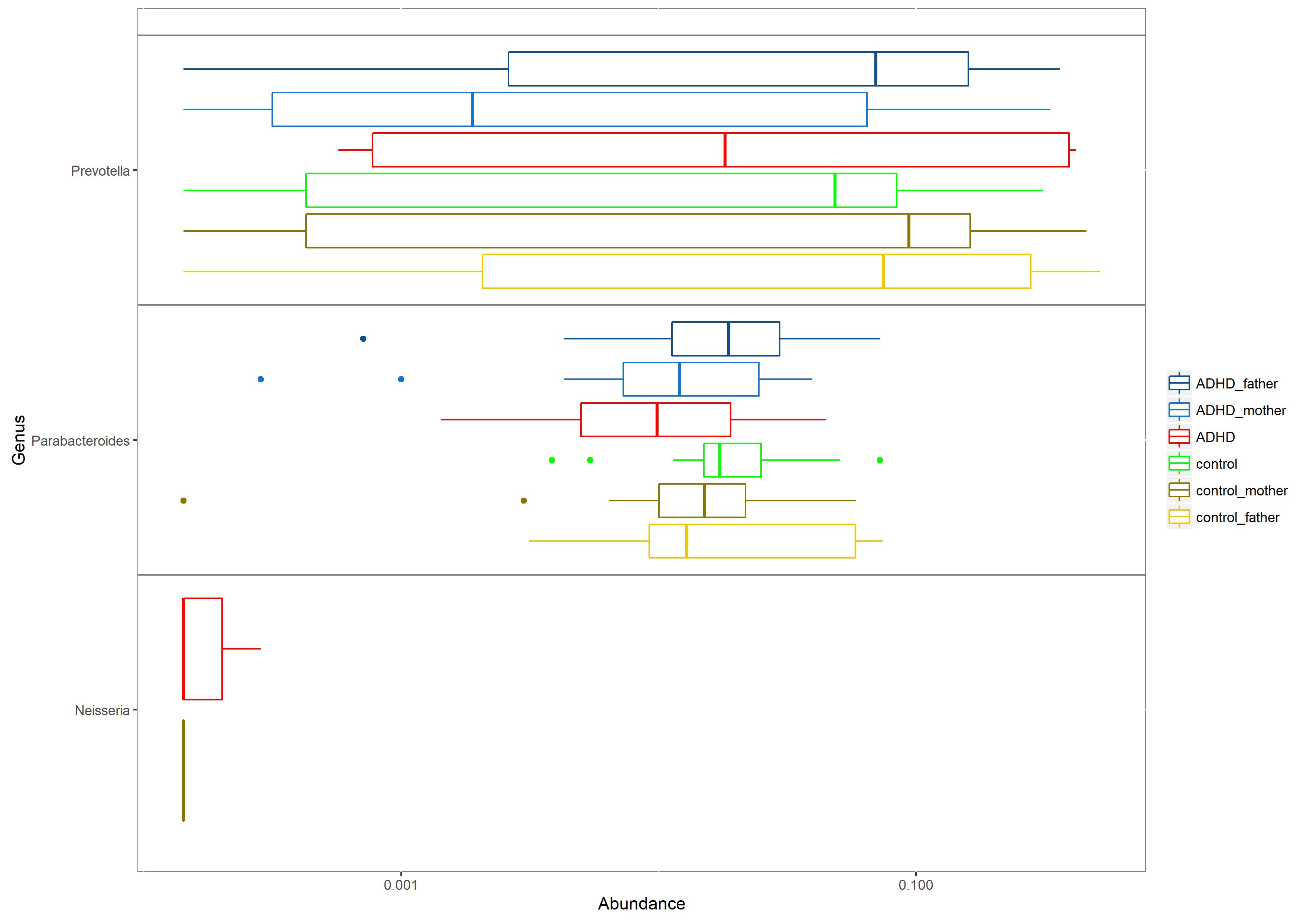

Supplement: S13 Fig — Boxplot showing the abundance of significant bacterial genera found by LEfSe analysis participants and their parents; IP, index patients; control, healthy controls. (JPEG) [file pone.0200728.s013.jpeg]

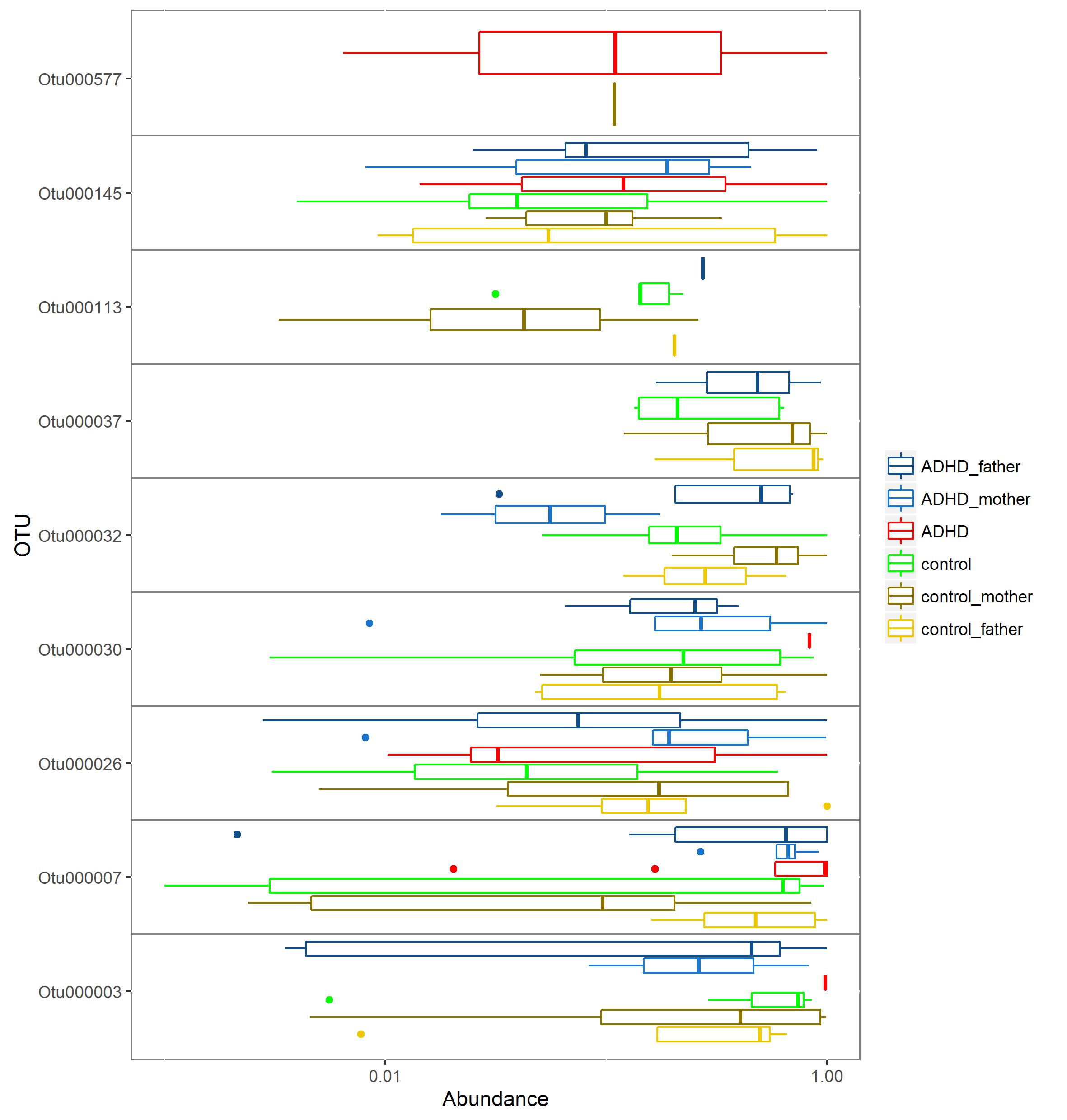

Supplement: S14 Fig — Boxplot showing the abundance of significant bacterial OTUs found by LEfSe analysis for participants and their parents; IP, index patients; control, healthy controls. (JPEG) [file pone.0200728.s014.jpeg]
