# Supplementary material for: Reduced microbiome alpha diversity in young patients with ADHD
Source: PLoS One. 2018 Jul 12;13(7):e0200728. doi: 10.1371/journal.pone.0200728 (PMC6042771; doi:10.1371/journal.pone.0200728)
Supplement: S1 Table — (DOCX) [file pone.0200728.s015.docx]

|  | **Shannon-Diversity-Indices** | | | **Observed species** | | |
| --- | --- | --- | --- | --- | --- | --- |
|  | **IP_mother** | **mother** | **IP_father** | **IP_mother** | **mother** | **IP_father** |
| **mother** | 0.029 | - | - | 0.029 | - | - |
| **IP_father** | 0.092 | 0.910 | - | 0.092 | 0.910 | - |
| **father** | 0.261 | 0.261 | 0.261 | 0.261 | 0.261 | 0.261 |

**S1 Table. Comparison of parental alpha diversity using pairwise Wilcoxon rank-sum tests.** Differences in alpha-diversity were tested using Wilcoxon rank-sum test. Alpha diversity from samples of parents from ADHD children [IP_mother (n =14), IP_father (n=10)] were tested against parents from control children [mother (n = 17), father (n = 12)].
