# Supplementary material for: Reduced microbiome alpha diversity in young patients with ADHD
Source: PLoS One. 2018 Jul 12;13(7):e0200728. doi: 10.1371/journal.pone.0200728 (PMC6042771; doi:10.1371/journal.pone.0200728)
Supplement: S2 Table — (DOCX) [file pone.0200728.s016.docx]

|  | **Shannon-Diversity-Indices** | | **Observed species** | |
| --- | --- | --- | --- | --- |
|  | **Controls** | **MPH** | **Controls** | **MPH** |
| **MPH** | 0.076 | - | 0.62 | - |
| **no_MPH** | 0.19 | 0.95 | 0.30 | 0.62 |

**S2 Table: Comparison of alpha diversity regarding medication using pairwise Wilcoxon rank-sum tests.** Differences in alpha-diversity were tested using Wilcoxon rank-sum test. Alpha diversity from samples of ADHD children with medication [MPH (n = 10)] were tested against controls (n = 17) and ADHD children without medication [no_MPH (n = 4)].
